# Optimization of the Vacuum Microwave Drying of Tilapia Fillets Using Response Surface Analysis

**DOI:** 10.3390/foods14050873

**Published:** 2025-03-04

**Authors:** Jianwen Ruan, Guang Xue, Yan Liu, Biao Ye, Min Li, Qing Xu

**Affiliations:** College of Ocean Engineering and Energy, Guangdong Ocean University, Zhanjiang 524088, China; ruanjw@gdou.edu.cn (J.R.); 15018336530@163.com (G.X.); liuyanz@163.com (Y.L.); yebiao1030@163.com (B.Y.)

**Keywords:** tilapia fillet, vacuum microwave drying (VMD), microwave power, vacuum degree, response surface optimization

## Abstract

This study looked at how vacuum microwave drying (VMD) affects the quality of tilapia fillets (*Oreochromis* spp.). It focused on the impact of fillet thickness, microwave power, and vacuum pressure on key quality parameters, such as water activity (Aw), texture, rehydration rate, and whiteness. A series of experiments were conducted with varying fillet thickness (3–7 mm), microwave power (132–396 W), and vacuum pressure (0.03–0.07 MPa) using a Box-Behnken design to optimize drying conditions. The findings revealed that fillets with a thickness of 3 mm had the lowest Aw and the highest hardness, while 7 mm thick fillets had the best rehydration rate, elasticity, and whiteness. Additionally, increasing microwave power caused a gradual decrease in Aw and whiteness, while elasticity, hardness, and the rehydration rate initially increased and then decreased. As vacuum pressure increased, Aw decreased, and both whiteness and elasticity improved. The optimal drying conditions for tilapia fillets were identified as 7 mm thickness, 330 W microwave power, and 0.06 MPa vacuum pressure. Under these conditions, the dried fillets achieved a comprehensive quality score of 93.94. The regression model developed for optimization showed strong predictive performance, with a minimal deviation of only 1.45% from the experimental results, indicating its reliability for predicting drying effects.

## 1. Introduction

Tilapia, renowned for its palatability and nutritional value, has gained widespread popularity among consumers. However, the high moisture content (78–80%) of fresh tilapia fillets renders them susceptible to enzymatic and microbial degradation [1]. Drying has emerged as an effective preservation method to extend the shelf life of aquatic products. Current drying technologies for fish fillets encompass heat pump drying [2], hot air drying [3], microwave drying [4], and vacuum freeze drying [5]. Heat pump drying, characterized by its energy efficiency, utilizes latent heat from water vapor during the drying process, minimizing heat loss. Despite its energy-saving advantages, this method exhibits limitations, including slow drying rates, substantial equipment investment, restricted applicability, and stringent environmental humidity control requirements [6]. Conversely, hot air drying suffers from inherent drawbacks, such as low drying efficiency, limited automation, slow heat transfer, significant degradation of heat-sensitive components, and inferior product quality [7]. Vacuum freeze drying effectively preserves color, aroma, flavor, nutrients, and bioactive components, while inhibiting microbial growth and maintaining enzymatic activity [8]. However, it is limited by high capital investment, operational costs, discontinuous processing, significant energy consumption, and elevated product prices [9,10]. To overcome the limitations of individual drying technologies, researchers have increasingly adopted combined drying approaches [11,12,13]. For instance, the integration of heat pump and vacuum freeze-drying technologies for shiitake mushrooms resulted in a 37.69% reduction in energy consumption compared to standalone vacuum freeze drying, while significantly enhancing product quality relative to heat pump drying [14]. Similarly, Antarctic krill processed using this combined approach demonstrated comparable quality to vacuum freeze-dried products, with a 50% reduction in drying time compared to individual methods [15]. Vacuum microwave drying (VMD) technology capitalizes on the reduced boiling point of water under vacuum conditions [16], facilitating simultaneous heat and mass transfer. This dual mechanism enables rapid drying, superior product quality, and enhanced process control [17]. The optimization of food drying processes emphasizes two critical parameters: product quality and energy efficiency. Recent studies have demonstrated the efficacy of combining osmotic dehydration with VMD in food applications [18,19,20].

Building upon these advancements, the integration of ultrasound-assisted osmotic treatment with VMD synergistically combines the benefits of both technologies. However, the application of this combined approach in seafood processing remains underexplored. This study focuses on tilapia fillets as the experimental material, employing color, texture characteristics, and rehydration rate as primary quality indicators. The drying process was evaluated based on two critical control parameters: moisture content and water activity (Aw). A systematic investigation was conducted using a single-factor circulation experiment, examining the effects of fillet thickness, microwave power, and vacuum level. These experimental results were used to establish a comprehensive quality score model, correlating processing parameters with product quality. Using this model, the key factors influencing VMD were analyzed, enabling the determination of optimal processing conditions for tilapia fillets. The developed regression model demonstrated stability and good fit, providing valuable theoretical guidance for parameter selection and process design in VMD of marine fish fillets and similar aquatic products.

## 2. Materials and Methods

### 2.1. Test Materials and Reagents

The experimental materials were purchased from Huguang Market, Zhanjiang, China. Sodium chloride (purity 99.5%) was purchased from Zhanjiang Keming Technology Co., Ltd., Zhanjiang, China. Upon purchase, the tilapias were transported to the laboratory within one hour and temporarily immersed in oxygenated water to maintain freshness. Following the Guidelines for the Humane Treatment of Experimental Animals (2006) issued by the Ministry of Science and Technology of China, the fish were humanely euthanized by rapid immersion in ice water. Subsequently, the tilapia, weighing (1.4 ± 0.2) kg, were processed by peeling, decapitation, evisceration, and slicing into 100 mm × 50 mm pieces of varying thicknesses for drying. The initial moisture content of tilapia measured using the oven method was 78~80% (wet base).

### 2.2. Experimental Design

**Figure 1:**
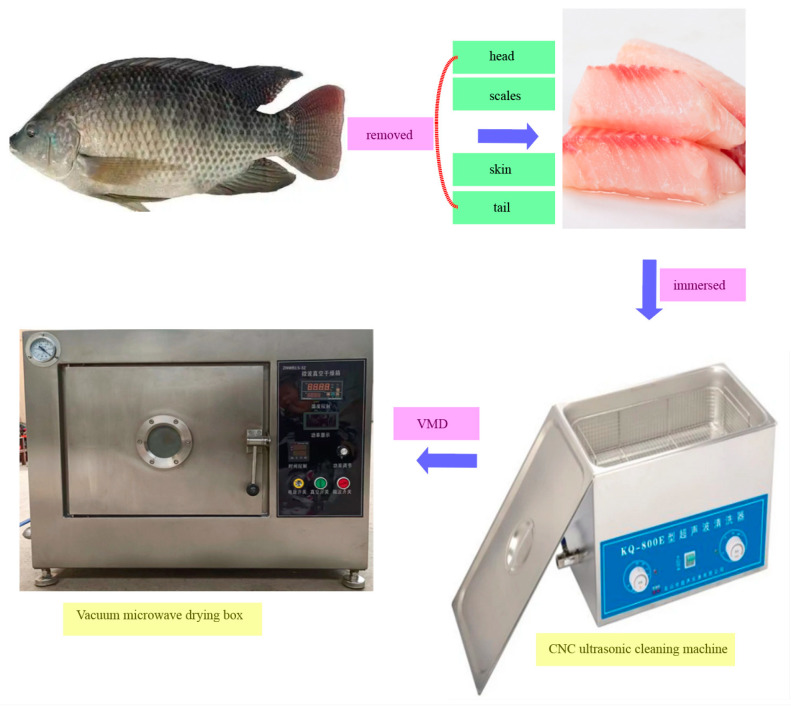
Schematic diagram of the vacuum microwave drying instrument.

It is recommended to conduct single-factor experiments on the experimental samples first, followed by response surface experiments, in order to comprehensively and efficiently optimize the experimental conditions and deeply analyze the interaction effects between multiple factors. The fish fillets were immersed in a 3.5% sodium chloride solution and subjected to ultrasonic-assisted permeation at 30 ± 0.5 °C in a water bath for 75 min, with a power of 400 W (CNC ultrasonic cleaning machine: KQ-500DE type, Kunshan Ultrasonic Instrument Co., Ltd., Kunshan, China). After treatment, excess surface moisture was removed using absorbent paper, and the fillets were transferred to a VMD oven (Model: JDH-1GZ, Guangzhou Yongze Microwave Energy Equipment Co., Guangzhou, China). The drying process alternates between 1 min of microwave vacuum and 2 min of vacuum operation, with the oven maintained at 30 ± 0.5 °C (Figure 1). The sample tray is preheated to minimize thermal inertia effects. The total drying time is 30 min. Each experiment was conducted in triplicate, and untreated samples served as the control group, ensuring consistent and reproducible results for evaluating the effects of VMD on tilapia fillets. The specific single factor experimental factors and horizontal parameter settings are shown in Table 1.

According to the results of single factor test, the response surface test scheme with three factors and three levels is optimized, and the test scheme is shown in Table 2.

### 2.3. Determination Method of Indicators

#### 2.3.1. Determination of Initial Moisture Content of Materials

The experimental moisture ratio ($w_{0}$) of tilapia were calculated using the Equation (1), with some modifications based on the work of Yilmaz et al. [4] as follows:

(1) $$w_{0}=\frac{m_{0}-m_{1}}{m_{0}}$$

where $w_{0}$ is the initial moisture content of fresh materials (g/g), $m_{0}$ is the mass of fresh materials (g), and $m_{1}$ is the mass of the absolutely dry material (g).

The dry basis moisture content of materials (AY120, Shimadzu Corporation, Kyoto, Japan with a resolution of 0.0001 g) is calculated using Equation (2):

(2) $$w=\frac{m-m_{0}\times \left(1-w_{0}\right)}{m_{0}\times \left(1-w_{0}\right)}$$

where w is the dry basis moisture content of the material, m is the mass g of the material at the end of single drying (g), $m_{0}$ is the fresh weight of the material (g), $w_{0}$ is the initial moisture content of fresh materials (g/g).

#### 2.3.2. Determination of Rehydration Rate

The rehydration rate is a crucial quality benchmark for dehydrated products, offering key insights into the water absorption capacity and the reversible structural recovery during the rehydration process. This parameter was determined according to the method described in the literature [21] with modifications. Briefly, the dried samples were immersed in a temperature-controlled water bath maintained at 40 ± 0.5 °C for 1 h. After immersion, the samples were removed and gently blotted with filter paper to remove excess surface moisture. The rehydrated mass of each sample was then measured using a precision electronic balance (accuracy: ±0.001 g). The rehydration rate ($R_{f}$) was calculated using the following equation:

(3) $$R_{f}=\frac{m_{f}-m}{m}\times 100\%$$

where $R_{f}$ is the rehydration rate of dry materials (%), $m_{f}$ is the mass of the dry material after rehydration (g), and m is the dry material mass (g).

#### 2.3.3. Whiteness Value Determination

The whiteness value is one of the important indexes to evaluate the quality of material products. The whiteness value was determined according to the method described in the literature [22] with modifications. Quantifying the chromaticity with the help of a colorimeter is beneficial to accurately judge the color change of materials. Measure the color data ($L^{*},$ $a^{*}$, $b^{*}$) of dry materials using a hand-held color difference meter, and calculate the whiteness value using Equation (4):

(4) $$w_{t}=100-\sqrt{\left[{\left(100-L^{*}\right)}^{2}+{a^{*}}^{2}+{b^{*}}^{2}\right]}$$

where $L^{*}$ indicates lightness value, and the greater the value, the whiter the material; $a^{*}$ indicates a red-green value, where a positive value indicates a reddish color, and a negative value indicates a greenish color; and $b^{*}$ indicates yellow and blue values, where positive values indicate yellow, and negative values indicate blue.

#### 2.3.4. Determination of Aw

The Aw value was determined according to the method described in the literature [23] with modifications. The Aw of dried materials was determined using an intelligent Aw meter (HD-4, Wuxi Huake Instrument Co., Wuxi, China).

#### 2.3.5. Determination of Moisture Content

The moisture content was determined according to the method described in the literature [24] with modifications. Determination of moisture in food (direct drying method) was performed to determine the moisture content of dry materials.

#### 2.3.6. Determination of Hardness and Elasticity

The texture of rehydrated fillets was determined using a texture analyzer (TMS-Pro, FTC Corporation, Washington, DC, USA). A cylindrical flat-bottomed probe (diameter 5 mm) was used, and the test conditions were set as follows: deformation 50%, test rate 60 mm/min, interval residence time 5 s, test distance 12 mm, compression mode as the test type, and each sample was tested 3 times.

#### 2.3.7. Determination of Calculation Method for Comprehensive Weighted Score of Indicators

The influence of three factors on the comprehensive quality of dried tilapia fillets was investigated using the comprehensive score method. The comprehensive weighted score value was determined according to the method described in the literature [25,26,27,28] with modifications. The full score is 100 points. Whiteness, Aw, moisture content, hardness, elasticity, and the rehydration rate are taken as quality characteristic indexes, and the weights of each index are 25 points, 20 points, 20 points, 10 points, 10 points, and 15 points, respectively. Among them, the greater the whiteness value, elasticity, and rehydration rate are, the better and the smaller the Aw, moisture content, and hardness. By calculating the membership degree of each index and combining it with the corresponding weight, the quality characteristic score of dry products is obtained.

The larger the measured value is, the better the whiteness value, elasticity, and rehydration rate are, as calculated as shown in Equation (5). The smaller the measured value is, the better the calculation of Aw, moisture content, and hardness, as shown in Equation (6):

(5) $$y_{1}=a\frac{s_{1}}{s_{0}}$$

(6) $$y_{2}=a\frac{s_{0}}{s_{1}}$$

where $y_{1}$, $y_{2}$ are the weighted score of indexes, a is an index, $s_{0}$ is used to determine the best value of an index in this group of experiments, and $s_{1}$ used to determine the experimental measured values of an index. The scores were compared with the sum of the scores of each index in this experiment.

### 2.4. Data Processing and Analysis

Data analysis was performed using SPSS Statistics 22.0 software, and significance was assessed using the least significant difference (Duncan) method. A *p*-value of <0.05 was considered statistically significant. The drawing software of origin_2022 was used for drawing, and the DESIGN-EXPERT 13 variance analysis platform and response surface panel analysis platform were used for data processing and analysis, and the significance analysis level was *p* (0.05).

## 3. Results and Discussion

### 3.1. Analysis of Single-Factor Experimental Results

#### 3.1.1. Effect of Thickness

**Figure 2:**
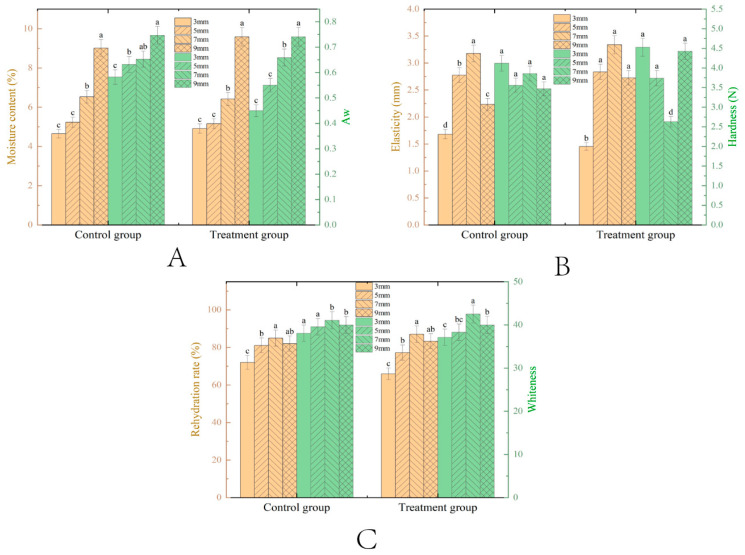
Changes in moisture content ((**A**), %) and, Aw (**A**); elasticity ((**B**), mm) and, hardness ((**B**), N); rehydration ((**C**), %) and whiteness (**C**) of tilapia fillet samples with different thickness conditions after VMD. The means between drying methods on the same thickness with different lowercase letters (a–d) differ significantly (*p* < 0.05).

Under pretreatment conditions with a fixed microwave power of 264 W and a vacuum degree of 0.05 MPa, the influence of different slice thicknesses on the drying characteristics of VMD samples was explored. Figure 2 shows the moisture content, Aw, elasticity, hardness, rehydration rate and whiteness of tilapia fillets with different slice thicknesses after VMD. As can be seen from Figure 2A, there is a significant difference between groups (*p* < 0.05), and there is a significant difference between the 3 mm and 9 mm groups. As the thickness of the pretreated tilapia fillets increased, the equilibrium moisture content gradually increased but remained within the safe range for dried products (10–15%) [1,29,30]. This can be attributed to several factors. For example, thicker fillets, due to lower microwave power density and longer moisture migration paths, hindered internal water removal. Additionally, thicker samples contain higher hydrophilic substances that interact with proteins (e.g., myofibrils) to form a dense gel network, trapping moisture within its 3D structure [31,32]. The reduced surface area-to-volume ratio in thicker fillets also led to reduced heat transfer efficiency, causing uneven moisture distribution with a soft interior and hard exterior, which further retained internal moisture [20,33]. In contrast, the 3 mm thick pretreated group had the lowest moisture content, likely because thinner fillets absorbed more microwave energy, efficiently removing both intermuscular and intracellular water. Aw, which indicates the strength of moisture binding, significantly differed among the groups. Pretreated groups showed lower Aw than the controls at all thicknesses, possibly due to the longer heating times required for thicker fillets, which slowed moisture migration. Furthermore, the pretreatment altered water distribution, increasing the amount of bound water trapped in cellular structures, which reduced Aw and extended the shelf life of the product [29,30].

As shown in Figure 2B, both control and treated groups displayed an initial increase followed by a decrease in elasticity. The 3 mm treated group showed the poorest elasticity, likely due to rapid microwave vacuum drying forming a loose, porous structure that collapsed under external pressure [34,35]. Conversely, the 7 mm group achieved optimal elasticity, attributed to larger honeycomb-like structures formed by cell rupture and shrinkage during drying. In hardness analysis, fillets initially softened then hardened with increasing thickness. Thinner samples (e.g., 3 mm) dried faster, causing severe shrinkage, pore deformation, and protein denaturation (e.g., myofibrillar protein coagulation), leading to surface hardening and higher hardness [36]. Samples with thicknesses (e.g., 5–7 mm) retained internal moisture, creating a humid microenvironment that enhanced toughness and reduced hardness. However, the 9 mm group unexpectedly hardened due to prolonged drying. Specifically, uniform pore contraction, increased density, and obstructed moisture channels under sustained high-temperature conditions reversed the softening trend.

The rehydration rate, which reflects the extent of structural damage in dried products, was significantly lower for the 3 mm thick pretreated tilapia fillets (Figure 2C). This is due to rapid heating causing cell wall rupture and the formation of dense myofibrillar structures, which hinder water reabsorption. Additionally, protein denaturation, including the oxidation of hydrophobic groups, further reduced the rehydration rate [37,38]. Both control and pretreated groups exhibited a rehydration rate trend that peaked and then declined as thickness increased. Initially (3–7 mm), the rehydration rate improved due to slower drying rates, which reduced structural stress and facilitated better microwave energy absorption, leading to the formation of more porous tissue [39]. However, with further increases in thickness (7–9 mm), the rehydration rate declined as microwave energy absorption decreased, weakening the vapor pressure-driven expansion. Notably, the 7–9 mm pretreated fillets outperformed controls due to enhanced permeability, whereas thinner samples (3–5 mm) benefited from the microwave-driven moisture migration, which outweighed the detrimental effects of cell damage caused by pretreatment. In terms of color, as a critical sensory attribute influenced by myoglobin derivatives and Maillard browning, the whiteness index peaked at intermediate thicknesses. Thinner fillets retained higher whiteness because the rapid drying suppressed the Maillard reaction. In contrast, thicker samples (e.g., 9 mm) darkened due to prolonged exposure to high temperatures and moisture, which accelerated browning [34].

#### 3.1.2. Effect of Power

**Figure 3:**
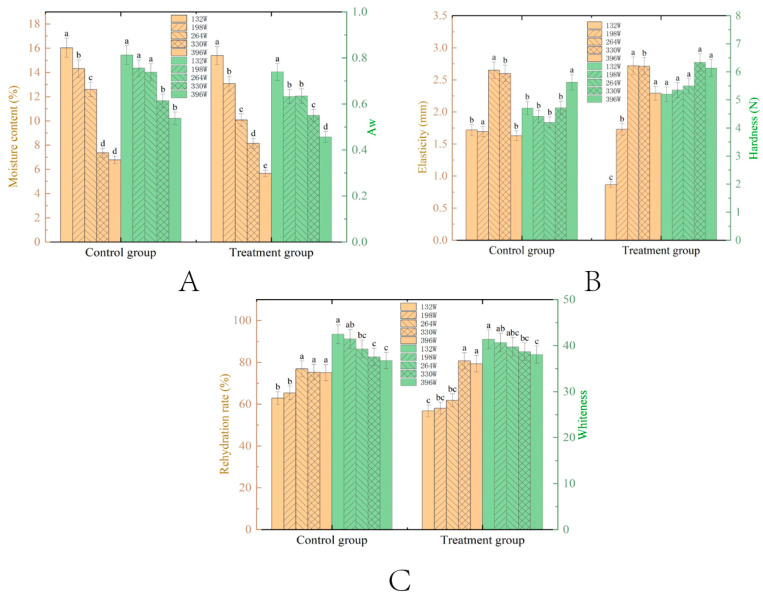
Changes in moisture content ((**A**), %) and Aw (**A**); elasticity ((**B**), mm) and hardness ((**B**), N); rehydration ((**C**), %) and whiteness (**C**) of tilapia fillet samples with different thickness conditions after VMD. The means between drying methods under the same power conditions with different lowercase letters (a–d) differ significantly (*p* < 0.05).

Pretreatment conditions were set at a fixed thickness of 7 mm and a vacuum degree of 0.05 MPa to investigate the effects of varying slice thicknesses on the drying characteristics of VMD samples. Figure 3A shows that moisture content and Aw of tilapia fillets subject to VMD decreased with higher microwave power, with pretreated groups consistently exhibiting lower Aw than controls (*p* < 0.05). This indicates stronger water-molecule binding in pretreated samples under high power, enhancing storability. Sodium chloride pretreatment dissociates into ions, adsorbing onto proteins to convert free water into bound water, further reducing Aw [40]. According to Figure 3A, pretreated groups generally had lower moisture content than controls (except at 330 W), attributed to enhanced microwave absorption and salt-soluble protein dissolution by NaCl, reducing water retention [41,42]. Higher power intensified structural damage (e.g., myofiber shrinkage, membrane rupture), weakening water-binding capacity and accelerating moisture loss. Moisture content and Aw were positively correlated. To balance drying efficiency and quality, higher microwave power is recommended for tilapia processing.

The textural properties (hardness and elasticity) of dried meat products, which are crucial for sensory evaluation, are influenced by factors such as protein hydration, fat content, and structural integrity. As shown in Figure 3B, pretreated samples demonstrated a peak-then-decline trend in hardness with increasing microwave power, consistently outperforming the controls (*p* < 0.05 at 264 W, 330 W, and 396 W). The initial increase in hardness (up to 330 W) was attributed to rapid water loss and the compaction of protein peptide chains, resulting in the formation of dense surface structures [43]. However, at 396 W, excessive dehydration disrupted the protein–water balance, causing the myofibers to loosen and ultimately reducing hardness. Elasticity followed a similar pattern, peaking at moderate power (e.g., 330 W), as shorter drying times helped preserve cellular integrity and promoted the formation of expanded honeycomb-like structures. At lower power (e.g., 132 W), prolonged drying led to cell damage, impairing recovery. In contrast, excessive power (396 W) induced protein conformational changes, which permanently degraded elasticity.

The rehydration rate of tilapia fillets is closely linked to their structural integrity, with porous networks enhancing the rehydration rate and severely damaged tissues reducing it. As shown in Figure 3C, the rehydration rate initially increased and then decreased with rising microwave power, which aligns with the previous literature [25]. At higher power levels (e.g., 264–330 W), hydrophilic groups were exposed, and flash evaporation occurred, creating porous structures that improved the rehydration rate [44]. However, at 396 W, surface charring and the development of a “hard exterior-soft interior” texture hindered water absorption, resulting in a reduced rehydration rate [19]. Whiteness, influenced by L* (moisture/protein denaturation), a* (myoglobin content), and b* (lipid oxidation), decreased with increasing power. Intense heat accelerated the migration of internal compounds to the surface and enhanced Maillard browning, which degraded the visual quality of the fillets [45]. Achieving an optimal microwave power that balances rehydration rate and whiteness is crucial for obtaining a desirable product appearance.

#### 3.1.3. Effect of Vacuum

**Figure 4:**
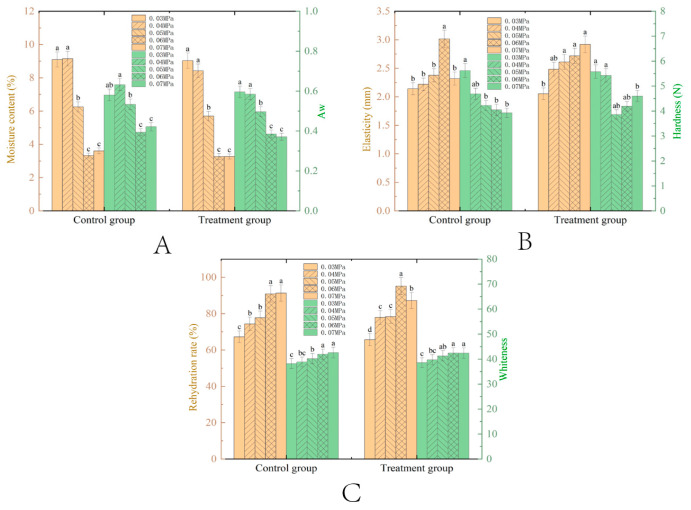
Changes in moisture content ((**A**), %) and Aw (**A**); elasticity ((**B**), mm) and hardness ((**B**), N); rehydration ((**C**), %) and whiteness (**C**) of tilapia fillet samples with different thickness conditions after VMD. The means between drying methods under the same vacuum conditions with different lowercase letters (a–c) differ significantly (*p* < 0.05).

The pretreatment conditions, including a fixed microwave power of 396 W and a thickness of 7 mm, were used to examine the impact of varying slice thicknesses on the drying characteristics of VMD samples. As shown in Figure 4A, the moisture content and Aw of pretreated tilapia fillets decreased with increasing vacuum levels during drying. Pretreated groups, except for those at 0.03 MPa, exhibited lower moisture content than the controls, likely due to NaCl infusion, which enhanced hydrophilicity and facilitated the removal of free water. Higher vacuum levels reduced water boiling points and improved mass transfer, resulting in lower final moisture content. At low vacuum (e.g., 0.03 MPa), surface shrinkage trapped internal moisture, while higher vacuum levels (≥0.06 MPa) reduced moisture content more effectively through larger pressure gradients and a reduced activation energy for water removal [44]. Notably, moisture content remained stable between 0.03–0.04 MPa (*p* > 0.05) and 0.06–0.07 MPa, indicating a critical vacuum threshold beyond which bound water became dominant, and further dehydration plateaued. Aw followed similar trends to moisture content, with lower vacuum levels correlating with higher Aw, due to the reduced mass transfer driving force.

Hardness and elasticity, key textural indicators of dried tilapia fillets, exhibited distinct responses to varying vacuum levels. As shown in Figure 4B, elasticity increased with higher vacuum levels due to accelerated moisture evaporation, reduced thermal degradation, and enhanced formation of porous network structures. Pretreated groups at lower vacuum levels (0.03–0.05 MPa) showed no significant differences, while higher vacuum levels (0.06–0.07 MPa) resulted in marked variations in elasticity, highlighting the dominant role of vacuum at extreme levels. Hardness initially decreased (0.03–0.05 MPa) as vacuum facilitated rapid moisture removal and puffing effects, leading to the creation of porous textures. However, it rebounded at 0.05–0.07 MPa due to structural shrinkage resulting from ultra-low moisture content and internal stress-induced disordered cellular arrangements, which narrowed the capillary pores.

Vacuum level critically influences the color and rehydration properties of tilapia fillets during microwave vacuum drying (Figure 4C). As the vacuum increased, the whiteness index initially rose, due to reduced Maillard browning and lipid oxidation resulting from lower boiling temperatures. However, at 0.07 MPa, the whiteness index declined, likely due to the migration of macromolecules to the surface under extreme vacuum conditions [31,32]. Lower vacuum levels (<0.06 MPa) promoted higher enzymatic activity and protein denaturation, while higher vacuum levels (>0.06 MPa) helped preserve color by limiting oxygen exposure. Significant differences between low and high vacuum levels (*p* < 0.05) confirmed the vacuum’s key role in color optimization. Regarding the rehydration rate, a peak-then-decline trend emerged with increasing vacuum. Moderate vacuum levels (0.04–0.06 MPa) enhanced the rehydration rate by creating porous structures through rapid drying-induced internal stress and moisture gradients. However, at 0.07 MPa, excessive vacuum caused damage to cellular networks, leading to a reduction in the rehydration rate [46]. Pretreated groups exhibited significant differences across vacuum levels (*p* < 0.05, except for 0.03 MPa), underscoring the dual role of vacuum in balancing structural integrity and drying efficiency.

### 3.2. Optimization of VMD Process Parameters of Tilapia Fillets and Its Influence on Quality

Using the factor level coding table in Table 2, the experimental design was carried out using BOX-Behnken, and the experimental scheme and results of tilapia fillets subject to VMD were established as shown in Table 3. Fifteen groups of experimental codes in the table were randomized, and each group of experiments was conducted in parallel for three times. Six indexes of tilapia fillets subject to VMD were statistically calculated by using the comprehensive quality scoring rule, and the total score was obtained. The higher the score, the better the quality.

DESIGN-EXPERT 13 was used to establish the regression model as shown in Equation (7) for the data in Table 3 (as shown below), and response surface regression analysis was used to process and analyze the data.

(7) $$Y=92.44+1.55X_{1}+2.28X_{2}+{1.28X}_{3}+0.64X_{1}X_{2}-0.32X_{1}X_{3}-1.92X_{2}X_{3}-4.94{X_{1}}^{2}-5.82{X_{2}}^{2}-9.43{X_{3}}^{2}$$

After the significance test of model Equation (7), the interaction items between $X_{1}$ and $X_{2}$, $X_{1}$ and $X_{3}$ are not significant (*p* > 0.05), and the optimized regression model is established after elimination.

The optimized regression model is obtained as Equation (8).

(8) $$Y=92.44+1.55X_{1}+2.28X_{2}+{1.28X}_{3}-1.92X_{2}X_{3}-4.94{X_{1}}^{2}-5.82{X_{2}}^{2}-9.43{X_{3}}^{2}$$

The significance test of model Equation (8) showed that all items in the optimized regression model significantly affected the comprehensive quality of dried tilapia fillets (*p* < 0.05). Therefore, each term can be retained in the model.

Model Equation (8) is used to predict the quality scores of 15 groups of dried tilapia fillets in Table 3, and the linear correlation analysis is performed. The results show that the predicted value of the total score has a good linear relationship with the actual value, with a determination coefficient R^2^ of 0.99 and a root mean square error (RMSE) of 0.943. It shows that the model can explain 99% of the changes in quality score based on thickness, microwave power and vacuum degree, so the model has high reference value.

The results of the significance test, determination coefficient, variance and mismatch analysis of the regression model show that the established regression model can be used to score the quality of tilapia fillets dried by vacuum microwave under different thickness, microwave powers and vacuum degrees. The optimized VMD process parameters are as follows: fillet thickness is 7 mm, microwave power is 330 W, and vacuum degree is 0.06 MPa.

#### 3.2.1. Intuitive Analysis of Thickness, Power and Vacuum Degree on the Quality Score of Tilapia Fillets Dried by Vacuum Microwave

##### Interactive Analysis of Quality Score of Fillets Dried by Vacuum Microwave

Because the three levels of material thickness, power and vacuum are all in the range of (−1~1) in response to the surface experiment, the primary and secondary relationship of each effect term on the quality score of tilapia fillets dried by vacuum microwave can be measured according to the absolute value of the coefficient of model 2. According to Table 3, the order of the influence of each effect term on the quality score is the quadratic term of $X_{3}$, the quadratic term of $X_{2}$, the quadratic term of $X_{1}$, the linear term of $X_{2}$, the interaction term between $X_{2}$ and $X_{3}$, the linear term of $X_{1}$ and the cubic term of $X_{3}$.

**Figure 5:**
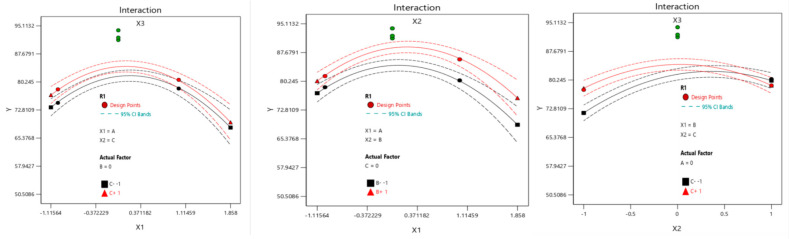
Visual interaction diagram of the quality score.

Interaction diagram can directly reflect the interaction between factors. In this experiment, Design-Expert 13 interactive descriptor platform is used to carry out inter-factor transactional analysis on the vested regression model (2). If there is no intersection between the vertical and horizontal between the two factors, it means that there is no interaction between the two factors. If there is intersection, it means that there is interaction between the two factors. As can be seen from Figure 5, there is a cross between $X_{2}$ and $X_{3}$, which shows that microwave power and vacuum degree have significant effects on the quality of dried tilapia fillets within the experimental range (*p* < 0.05).

##### Three-Dimensional Curved View of the Quality Score of Tilapia Fillets Dried by Vacuum Microwave

**Figure 6:**
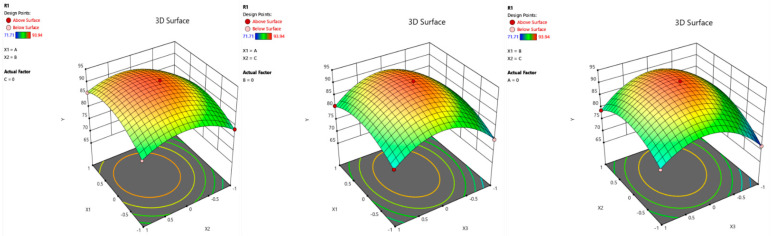
Intuitive quality score of the 3D surface.

In order to study the influence of material thickness $X_{1}$, microwave power $X_{2}$ and vacuum degree $X_{3}$ in the established model on the quality score of dried tilapia fillets, and fix the zero level of one of these factors, a three-dimensional surface drawing was made using the three-dimensional surface plotter platform of DESIGN-EXPERT 13, as shown in Figure 6. The three-dimensional curved surface can directly reflect the influence of various factors on the quality score of dried tilapia fillets.

#### 3.2.2. Parameter Optimization and Model Verification

**Figure 7:**
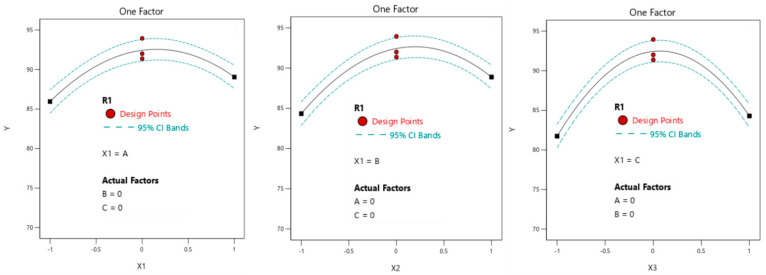
Optimal prediction of the VMD quality score of tilapia fillet.

The technological parameters of the quality score of tilapia fillets dried by vacuum microwave were optimized using the predictive characterization platform function of DESIGN-EXPERT 13, as shown in Figure 7. Figure 7 shows that within the scope of this experimental study, when $X_{1}$ = 0.17, $X_{12}$ = 0.2, and $X_{3}$ = 0.04, the predicted theoretical score of the quality of dried tilapia fillets is 92.82.

In order to verify the accuracy of the established model, and according to the requirements of experimental equipment and experimental operating conditions, the experimental parameters of fillet thickness, power, and vacuum degree are set to the zero level. Specifically, fillet thickness is 7 mm, microwave power is 330 W, and vacuum degree is 0.06 MPa. Under this condition, three parallel experiments were carried out. The experimental value is 91.47, and the relative error between the experimental value and the theoretical value is 1.45%, which shows that the model can predict the drying effect well. It shows that the optimization process has certain guiding significance.

Because the variables in the regression model Equation (8) are coded values, it is necessary to bring the mathematical expressions of coded values in Table 2 into the model. This includes the actual mathematical expressions of the quality score y of tilapia fillets dried by vacuum microwave and the fillet thickness $Z_{1}$, microwave power $Z_{2}$, and vacuum $Z_{3}$ as shown in Equation (9):

(9) $$Y=67.32-59.74Z_{1}+0.21Z_{2}+{202Z}_{3}-1.24{Z_{1}}^{2}-0.0036{Z_{2}}^{2}-94300{Z_{3}}^{2}-Z_{2}Z_{3}$$

## 4. Conclusions

The quality of tilapia fillets subject to VMD is influenced by fillet thickness, microwave power, and vacuum level. Thinner fillets (3 mm) exhibit lower Aw and higher hardness, while thicker fillets (7 mm) show better elasticity, rehydration rate, and whiteness. Optimal conditions were identified as 7 mm thickness, 330 W microwave power, and 0.06 MPa vacuum level, yielding a quality score of 92.82 with minimal deviation (1.45%). These findings, applicable to other seafood, highlight VMD’s advantages over traditional methods, including improved nutrient retention, texture, color, and energy efficiency, offering valuable insights for industrial-scale optimization and sustainability.

## Figures and Tables

**Table 1:** Single factor experimental factors and levels.

Factor	Values
Thickness T/(mm)	3, 5, 7, 9
Power W/(W)	132, 198, 264, 330, 396
Vacuum degree *V*/(MPa)	0.03, 0.04, 0.05, 0.06, 0.07

**Table 2:** Experimental scheme design of response surface optimization.

Factor	Thickness of Fish Fillet Z_1_/(mm)	Microwave Power Z_2_/(W)	Vacuum Degree Z_3_/(MPa)
level (+1)	9	396	0.07
level (0)	7	330	0.06
level (−1)	5	264	0.05
Changes in pitch (Δ*j*)	2	66	0.01
Coded value formula	X_1_ = (Z_1_ − 7)/2	X_2_ = (Z_2_ − 330)/66	X_3_ = (Z_3_ − 0.06)/0.01

**Table 3:** Response surface experimental design scheme and results.

No.	Thickness (X_1_)	Microwave Power (X_2_)	Vacuum Degree (X_3_)	Comprehensive Score
1	−1	−1	0	78.71
2	−1	1	0	81.6
3	1	−1	0	80.48
4	1	1	0	85.91
5	0	−1	−1	71.71
6	0	−1	1	77.71
7	0	1	−1	80.49
8	0	1	1	78.83
9	−1	0	−1	74.7
10	1	0	−1	78.5
11	−1	0	1	78.28
12	1	0	1	80.81
13	0	0	0	91.37
14	0	0	0	92
15	0	0	0	93.94

## Data Availability

The original contributions presented in this study are included in the article, and further inquiries can be directed to the corresponding authors.
